# Undersized stent graft for treatment of cephalic arch stenosis in arteriovenous hemodialysis access

**DOI:** 10.1038/s41598-020-69402-3

**Published:** 2020-07-27

**Authors:** Eric Po-Yu Huang, Ming-Feng Li, Chia-Chi Hsiao, Hsin-Yu Chen, Ping-An Wu, Huei-Lung Liang

**Affiliations:** 1 Department of Radiology, Kaohsiung Veterans General Hospital, No. 386, Ta-Chung 1st Road, Kaohsiung, 813 Taiwan, ROC; 2 Department of Medical Imaging, Hualien Buddhist Tzu-Chi General Hospital, 707, Sec. 3, Chung-Yang Rd, Hualien, 970 Taiwan, ROC; 3 Department of Medical Imaging and Radiology, Shu-Zen Junior College of Medicine and Management, No. 452, Huanqiu Road, Luzhu District, Kaohsiung City, 821 Taiwan, ROC; 4 School of Medicine, National Yang-Ming University, No. 155, Sec. 2, Linong Street, Taipei, 112 Taiwan, ROC; 5 Division of Nephrology, Department of Internal Medicine, Kaohsiung Veterans General Hospital, No. 386, Ta-Chung 1st Road, Kaohsiung, 813 Taiwan, ROC

**Keywords:** Outcomes research, Medical imaging, Quality of life

## Abstract

Cephalic arch stenosis (CAS) is a common cause of AV dialysis access failure and is notoriously difficult to treat with conventional venoplasty. Although stent graft (SG) placement has improved patency rate, they are prone to stent failure caused by edge stenosis. We investigate the effect of SG diameter relative to cephalic vein on patency rate among hemodialysis patients with dysfunctional arteriovenous access caused by CAS. We identified 22 patients with recalcitrant cephalic arch stenosis or post-venoplasty vessel rupture and received SG placement. Patients were divided into two groups based on the stent-to-vessel diameter (S/V) ratio: undersized group, < 1; and apposed group, 1–1.2. Outcomes were assessed through follow-up angiography. S/V ratio was significant smaller in the undersized patient group (*p* < 0.001). Placement of undersized SG demonstrated higher primary stent (*p* = 0.001) and access patency rates (*p* = 0.021) and a reduced number of post-treatment reinterventions per access year (*p* = 0.021). A decreased number of lateral edge stenosis was noted in undersized SG (*p* = 0.005). Increased S/V ratio was significantly associated with lateral edge stenosis (OR = 5.19; *p* = 0.027). Undersized SG is associated with higher primary stent and access patency rate, and decreased number of post-SG interventions, and are suggested in the treatment of cephalic arch stenosis.

## Introduction

Hemodialysis is the most common treatment for an increasing number of patients with end-stage renal disease. In most of these patients, the ideal circulation access point is through an arteriovenous fistula (AVF) or an arteriovenous graft (AVG) on the upper limb^1,2^. Despite being the ideal route, arteriovenous hemodialysis access (AVA) is still plagued by frequent dysfunction, with venous stenosis being the most common underlying culprit. Among patients with venous stenosis, cephalic arch stenosis (CAS) is a common cause of dialysis access failure with a reported incidence as high as 77% among patients with brachiocephalic AVFs^3,4^. This results in loss of dialysis access requiring angiographic or surgical treatment, and increases burden on the patient and healthcare providers.

While venoplasty is the primary option for treatment of most venous stenosis, it’s effectiveness on treatment of CAS is suboptimal and brief^5,6^. As venoplasty alone has not demonstrated significant lasting benefits for CAS, bare-metal stent (BMS) placement and stent graft (SG) have been prescribed with better but still variable results. A recent 2 year comparison between venoplasty, placement of BMS and placement of SG, has shown primary patency of 23.3, 52.2 and 82.7% at 6 months and 9.5, 12.9 and 44% at 12 months, respectively^7^.

Although SG has demonstrated significant benefits over conventional venoplasty and BMS at maintaining patency of vascular access, they are far from perfect and are prone to “edge stenosis”, defined by stenosis at the interface between SG and adjacent vessel wall, within 5 mm of its margin^8,9^. In a recent study, among all stent stenosis after SG treatment of CAS, 52% were caused by lateral edge (cephalic vein) stenosis. Medial edge (axillary vein) stenosis and in-stent stenosis occurred in 17%, and 2% respectively^10^.

**Figure 1:**
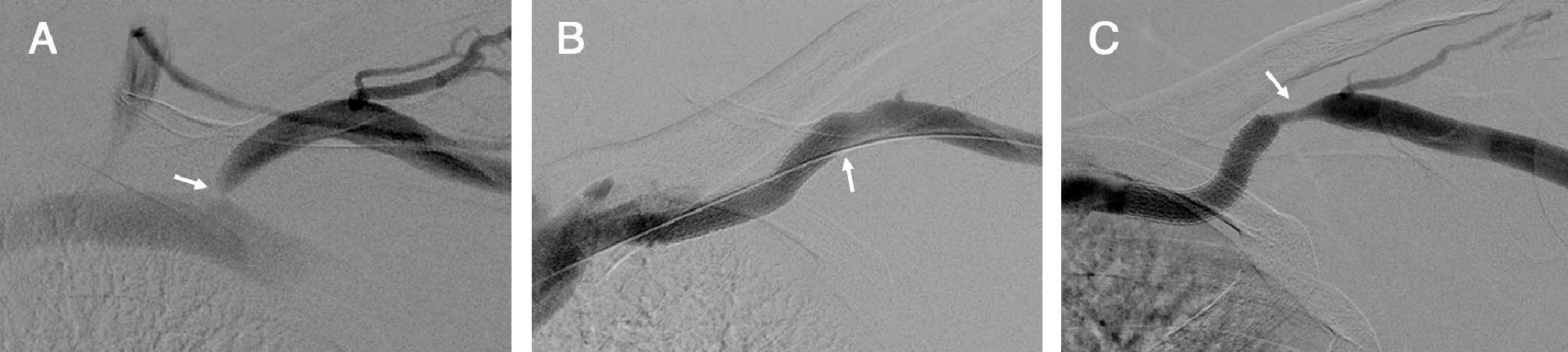
Initial venography and follow up venography of apposed stent graft in the cephalic arch. (**A**) Digital subtraction venography of left cephalic arch in a 32-year-old man with high venous pressures on dialysis. Imaging demonstrates significant recalcitrant stenosis (arrow). (**B**) Venography after successful 8 × 50 mm Viabahn Endoprothesis deployment and balloon dilatation at the cephalic arch, with apposition to the lateral cephalic vein (arrow) (**C**) 1 month follow up due to high venous pressure. Venography shows significant lateral edge stenosis (arrow).

**Figure 2:**
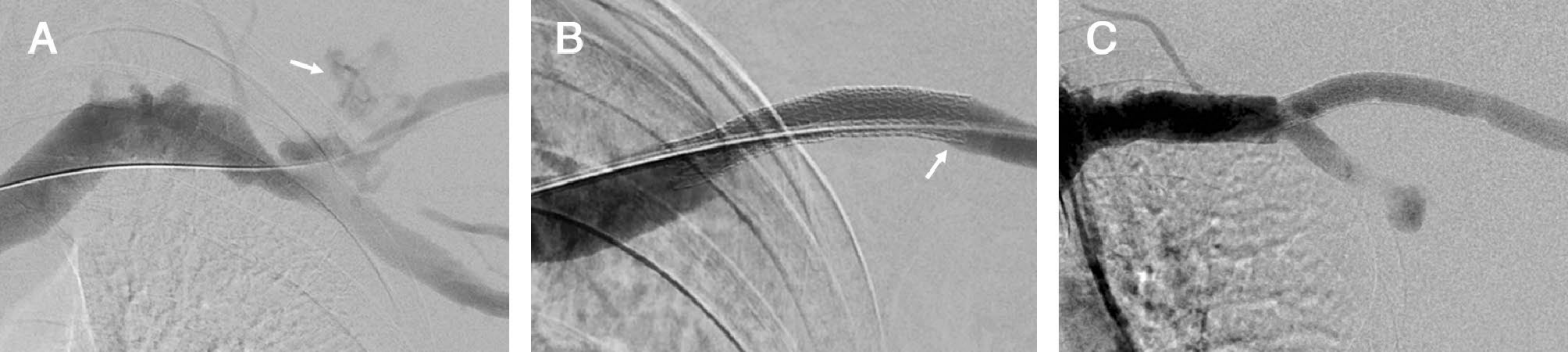
Initial venography and follow up venography of undersized stent graft in the cephalic arch. (**A**) Digital subtraction venography of left cephalic arch in a 66-year-old female presenting with high venous pressures on dialysis. Imaging demonstrates vessel rupture after balloon venoplasty (arrow). (**B**) Venography after successful 7 × 50 mm Viabahn Endoprothesis deployment and balloon dilatation at the cephalic arch, with an undersized SG. No apposition of the SG to the cephalic vein at the lateral edge (arrow) (**C**) most recent venography follow up shows no evidence of SG stenosis and no obstruction of the axillary venous return. No treatment within the SG area has been performed during this 4 year follow up period.

To the best of our knowledge, there have not been any previous studies discussing the optimal SG sizing for improving primary patency time in patients with cephalic arch stenosis. As a result, interventional radiologists continue to follow the guidelines found in the each stent graft manual, which recommend of oversizing SG by 5–20% of the original vessels^11^. In doing so, we noticed low SG patency rate with frequent reinterventions necessary to maintain patency (Fig. 1). On the other hand, we noticed higher patency rate in patients with SGs smaller than the diameter of the cephalic vein, requiring less reinterventions (Fig. 2). To confirm our observation, this study evaluated the effect of undersized SGs, relative to the adjacent cephalic vessel, on the clinical outcomes of patients with CAS.

## Materials and methods

### Patient population

From our vascular access database of a single institution, all patients with AVAs whom had been treated with SG placement at the cephalic arch between 2013 and 2019 were retrospectively identified. 22 patients (6 males, 16 females; mean age 68.4 yrs; range 32–86) were ultimately selected. All patients were treated by interventional radiologists with at least 7 years’ experience in treating AVA dysfunction. Indication for SG placement was recalcitrant stenosis, defined by intraprocedural elastic recoil or stenosis recurrence at least twice within a period of 3 months, of the CAS in 10 patients, and vessel rupture after venoplasty in 12. Demographic and clinical data were collected from the medical charts for this retrospective analysis. Informed consent was waived and this study was approved by the institutional review board of Kaohsiung Veterans General Hospital (IRB No. KSVGH20-CT3-13). All methods below were carried out in accordance with relevant guidelines and regulations.

**Figure 3:**
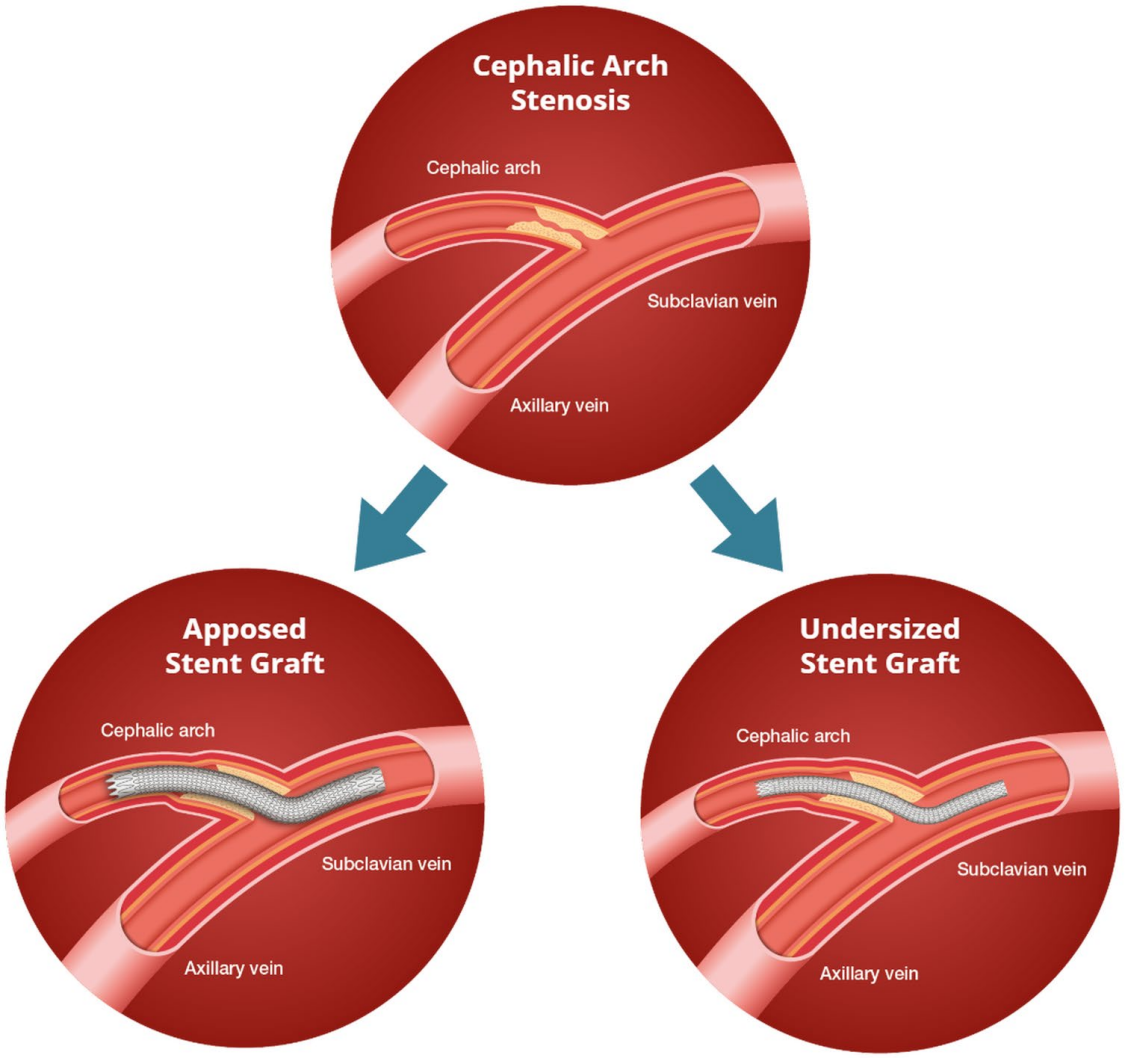
Depiction of undersized vs apposed SG placement at a cephalic arch. Undersized group: S/V ratio < 1 with SG apposed centrally at the stenosis location of the cephalic arch, and free-floating at the subclavian and distal cephalic vein. Apposed group: S/V ratio ranged from 1 to 1.2, with the SG apposed to the adjacent cephalic vein.

Patients were separated into two groups based on stent-to-vessel diameter ratio (S/V ratio) and SG apposition to the adjacent cephalic vessel. Patients were placed into the apposed subgroup if the S/V ratio ranged from 1 to 1.2. Patients in the undersized SG group had S/V ratio less than 1 and greater than 1 mm difference between lateral edge of the SG and cephalic vein. In both groups, SG remains apposed to the stenotic vessel at the central portion of the cephalic arch, thus preventing stent migration and allowing adequate extravasation control in patients with a ruptured vessel. The difference between the two groups lies at the lateral edge where the SG of undersized group is free-floating within the cephalic vein, versus the SG of the apposed group which is in contact with vessel wall of the adjacent cephalic vein. Depiction of undersized versus apposed SG is shown in Fig. 3.

### Technique

After venous access was obtained through puncture of outflow vein or graft, digital subtraction angiography was performed at the cephalic arch and CAS was identified. Intravenous Heparin during the procedure was given at the discretion of the attending interventional radiologist with doses up to 2,000 IU. Venoplasty was initially carried out with a high-pressure balloon. Viabahn Endoprosthesis (W. L. Gore & Associates, Inc; Flagstaff, Arizona) was placed for treatment of recurrent stenosis of celiac arch or vessel rupture following balloon dilatation at the CAS. Stent length was selected such that the SG covered the entire cephalic arch, the entire length of all diseased cephalic vein, and protruded into the subclavian vein. The diameter of the SG chosen was initially determined by the size of the adjacent cephalic vein following the guidelines stated in the SG manual^11^. Venoplasty within the SG was performed with similar sized balloon to ensure adequate deployment. Completion venography was performed, and hemostasis of venous wound was achieved via manual pressure. No post-procedural antithrombotic medications were prescribed for any patients.

### Measurements

All procedures were using one of two angiographic units (ArtisZee biplane and AXIOM Artis dTA, Siemens Healthcare, Forchheim, Germany). Measurement was performed using the built-in automated calibration system available on the Siemens angiographic unit (Syngo Software, Siemens Healthcare, Forchheim, Germany). Measurements of the vessel size and S/V ration were taken by an interventional radiologist with 7 years of experience treating AV access dysfunction (L.M.F.). All films and measurements were reviewed by our chief of interventional radiology with 30 years of experience (L.H.L.). Final decision in case of any discrepancy was reached by consensus between the two reviewers.

### Surveillance and follow-up

Surveillance was performed using diagnostic fistulography during follow-up venoplasty due to stenosis at cephalic arch or any other access circuit location. Diagnostic fistulography with or without venoplasty was also performed for any patients with symptoms of access dysfunction. Any evidence of stenosis (> 50%) associated with the cephalic arch SG or any other de novo access circuit stenosis was recorded. Primary stent patency, primary access patency, secondary patency, interventions per access-year after SG placement, and stenosis pattern of the SG were recorded for each patient during each examination. All patients were followed-up at least once post SG placement at the cephalic arch.

### Definitions

The S/V ratio was determined by calculating the ratio between the stent diameter and the vessel diameter at the adjacent cephalic vein on angiography. Technical success of SG placement was defined by adequate deployment and expansion of SG in the appropriate position, resolution of stenosis (residual stenosis < 30%) and/or resolution of contrast extravasation under angiography. Clinical success was defined by resumption of normal dialysis via AVAs and resolution of patient symptoms related to upper limb venous hypertension. Primary stent patency was defined as time interval from initial SG placement until intervention is required within the SG or within 5 mm of its edge. Primary access patency was defined as time interval from initial SG placement until next intervention, regardless of stenosis site. Secondary patency was defined as the time from the initial SG placement to the abandonment of the AVA^12^.

### Statistical analysis

Statistical analysis was performed using SPSS 22 (IBM Corp. Released 2013. IBM SPSS Statistics for Windows, Version 22.0. Armonk, NY: IBM Corp.) Patency over time was estimated using the Kaplan–Meier method, as recommended^12^. Log-rank statistics were used to compare survival curves, and hazard ratio (HR) was calculated through Cox proportional hazards regression analysis. Patient characteristics were calculated using the Student T test or Mann–Whitney test for continuous variables and Chi squared test for categorical variable. Intervention rate was calculated as the frequency of interventions for the duration of access patency during the 1-year follow-up period. To identify the risk factors associated with lateral edge stenosis, univariate analyses and multivariate logistic regression with variables selected in a stepwise backward manner were used, with results presented as odds ratios (ORs) and 95% confidence intervals (CIs). A *p* value < 0.05 was set as the threshold for significance.

## Results

### Patients

From 2013 to 2019, 22 patients were selected into this study. Patients in both groups had comparable clinical and demographic characteristics (Table 1). AVA was obtained through AVF in 17 patients, and through AVG in the remaining five. The mean follow-up time was 19.6 months (range, 2.5–51.6 months). Characteristics variables included age, sex, AV access location and type, years on dialysis, indication for venoplasty and SG placement, and the number of previous angioplasties. There was no significant difference between the two groups.

**Table 1:** Demographic and clinical characteristics for patients in the two study groups.

Characteristics	Apposed (*n* = 10)	Undersized (*n* = 12)	*p* value
Age (years)	66.3 ± 14.2	70.8 ± 10.9	0.488
Male/female	4/6	2/10	0.219
AVA type			0.201
Brachiocephalic fistula	5 (50)	10 (83.3)	
Radiocephalic fistula	2 (20)	0 (0)	
Loop AVG	2 (20)	1 (8.3)	
Straight AVG	1 (10)	1 (8.3)	
Location (right/left arm)	2/8	4/8	0.481
Year on dialysis	6.34 ± 8.84	5.35 ± 3.71	0.748
Indication for initial procedure			0.437
High venous pressure	7 (70)	9 (75)	
Arm swelling	0 (0)	1 (8.3)	
Thrombosis	3 (30)	2 (16.7)	
Indication of SG placement			0.431
Vessel rupture	5	7	
Recalcitrant stenosis	5	5	
Previous angioplasties	4.40 ± 4.55	8.58 ± 9.52	0.196

Continuous data are presented as the means ± standard deviation; categorical data are given as the counts (percentage).

*AVA* arteriovenous hemodialysis access, *AVG* arteriovenous graft, *SG* stent graft.

### Procedure characteristics and clinical outcomes (Table 2)

**Table 2:** Procedure characteristics and outcomes of SG placement in the two study groups.

Characteristics	Apposed (*n* = 10)	Undersized (*n* = 12)	*p* value
**Procedure variables**			
Technical success	10 (100)	12 (100)	1.00
Clinical success	10 (100)	12 (100)	1.00
SG Size (mm)			0.003*
5	0 (0)	1 (8.33)	
6	0 (0)	6 (50)	
7	7 (70)	5 (41.7)	
8	3 (30)	0 (0)	
Stent length (mm)			0.321
50	4 (40)	4 (33.3)	
70	2 (20)	0 (0)	
100	3 (30)	4 (33.3)	
150	1 (10)	2 (16.7)	
250	0 (0)	1 (8.33)	
300	0 (0)	1 (8.33)	
Cephalic arch stenosis (%)	86.3 ± 14.1	80.7 ± 13.2	0.290
S/V Ratio	1.11 ± 0.06	0.76 ± 0.08	< 0.001*
**Outcomes**			
Follow-up time (months)	20.0 ± 19.4	18.6 ± 14.2	0.931
Interventions per access-year after SG placement	4.20 ± 3.01	1.75 ± 2.68	0.021*
Restenosis pattern			0.005*
Lateral edge stenosis	7 (70)	2 (16.7)	
Medial edge stenosis	0 (0)	2 (16.7)	
Both edge stenosis	2 (20)	0 (0)	
Intra-stent stenosis	0 (0)	1 (8.33)	
Stent thrombosis	2 (20)	1 (8.33)	0.426

Continuous data are presented as the means ± standard deviation; categorical data are given as the counts (percentage).

*SG* stent graft, *S*/*V* ratio stent-to-vessel diameter ratio.

*p < 0.05.

Procedure characteristics and clinical outcomes are shown in Table 2. Technical and clinical success for SG placement was 100% after initial stent placement. Viabahn Endoprosthesis was used in all 22 patients. SG diameter ranged from 5 to 8 mm, and from 50 to 300 mm in length. Two patients had SG exceeding 150 mm due to severe extended stenosis of distal cephalic vein. To adequately support these stenotic vessels and preserve the AVA, a longer SG had to be chosen. Eventual stent stenosis after SG placement was noted in 14 of the 22 patients. Among these patients, lateral edge stenosis occurred in 9 patients, medial edge stenosis in 2 patients, both edge stenosis in 2 patients, and intra-stent stenosis in one patient. Three cases were ultimately complicated by thrombosis of stent graft, secondary to lateral edge stenosis, and occurred in patients within the apposed SG group. No stent fractures or other procedure-related complications of SG placement were noted during the study period. Three patients were lost to follow-up during the study period, along with 3 deaths, but none were procedure-related or occurred within 60 days of SG placement.

Clinical outcomes of SG placement between the apposed group and the undersized group is shown in Table 2. The size of SG used in the undersized patient group was significantly smaller than the size used in the apposed patient group (*p* = 0.003). The S/V ratio in the undersized group was significantly less than in the apposed patient group (1.11 ± 0.06 vs 0.76 ± 0.08, p < 0.001). After SG placement, the interventions per access year needed for AVA dysfunction after SG placement was significantly less in the group with undersized SG placement (1.75 ± 2.68 vs 4.20 ± 3.01, p = 0.021). Furthermore, stenosis site after SG placement was significantly different between the apposed group and the undersized group (p = 0.005). Other variables were shown to be comparable between the two study groups.

### Patency

Primary stent patency rate for all SGs were 71%, 56%, 34%, 34%, and 22% at 3,6,12, 24 and 30 months; Primary access patency rate for all SGs were 68%, 41%, 12%, 12%, and 0% at 3,6,12, 24 and 30 months. Secondary patency rate for access were 100%, 100%, 100%, 92% and 83% at 3,6,12, 24 and 30 months.

**Figure 4:**
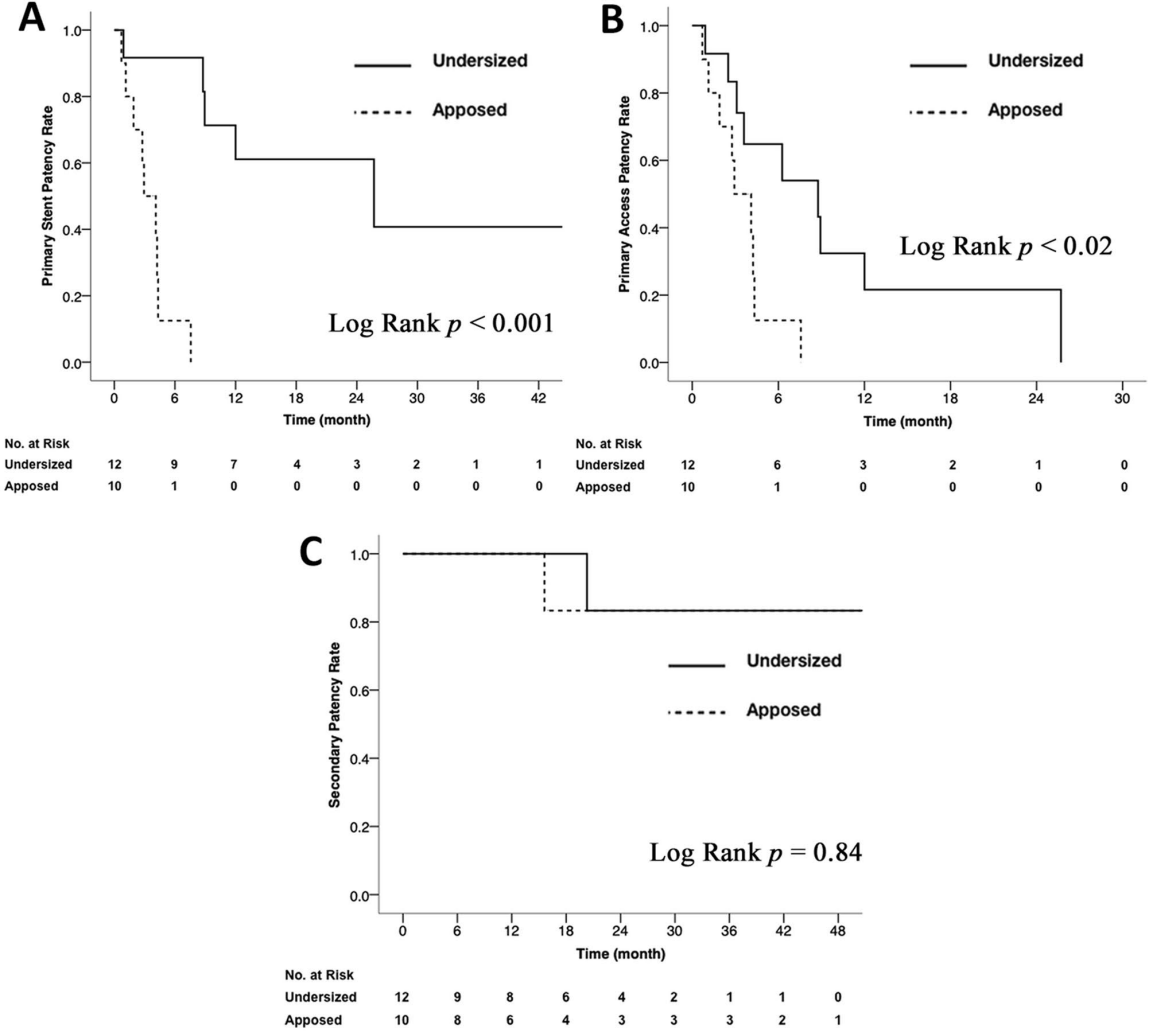
Kaplan–Meier curves for stent patency after stent graft placement for cephalic arch stenosis. (**A**) primary stent patency, (**B**) primary access patency, and (**C**) secondary access patency for patients after SG placement for CAS. *p* value derived by log-rank sum testing.

Primary stent patency for each SG, primary access rate, and secondary patency for each group is shown in Table 3. Primary stent patency rate for patients in the undersized subgroup showed significantly higher patency rate when compared to the apposed subgroup of patients (log-rank test: χ^2^ = 15.40, *p* < 0.001*; HR* = 0.04; 95% CI: 0.01–0.36) (Fig. 4A). This trend continued when comparing primary access patency between the two groups. Primary access patency rate was significantly higher among patients with undersized SG patients versus patients with apposed SG placement (log-rank test: χ^2^ = 5.30, *p* = 0.021; *HR* = 0.29; 95% CI: 0.09–0.88) (Fig. 4B). Secondary patency rate showed comparable patency rate when compared to the apposed subgroup of patients (log-rank test: χ^2^ = 0.04, *p* = 0.843; *HR* = 0.76; 95% CI: 0.05–12.15) (Fig. 4C).

**Table 3:** Primary stent, primary access and secondary access patency rate after stent placement.

Patency rate	Group	Months						*p* value
		1	3	6	12	24	30	
Primary stent (%)	Apposed	90	50	13	0	0	0	
	Undersized	92	92	92	61	61	41	< 0.001*
Primary access (%)	Apposed	90	50	13	0	0	0	
	Undersized	92	83	65	22	22	0	0.021*
Secondary (%)	Apposed	100	100	100	100	83	83	
	Undersized	100	100	100	100	83	83	0.843

*p < 0.05.

### Risk factors associated with lateral edge stenosis

The lateral edge stenosis, regardless of medial edge stenosis, was the most common pattern of stenosis (11/14; 78.6%) among patients with SG related stenosis. Therefore, the risk factors leading to lateral edge stenosis were analyzed. The univariate and multivariate analysis identified only increased S/V diameter ratio (*OR* = 5.19; 95%CI: 1.21–22.25; *p* = 0.027) was significantly associated with lateral edge stenosis.

## Discussion

Cephalic arch stenosis is a well-known location for stenosis among hemodialysis patients with AVAs, especially in patients with brachiocephalic fistulas^3,4^. The pathophysiology of CAS is still being investigated and various contributing factors have been suggested. Physical factors such as venous valves in the cephalic arch and the traversal of the cephalic arch through the deltopectoral groove may constrict the blood vessel and restrict venous return into the cephalic vein. Fluid dynamic changes as a result of the curvature as the cephalic arch enters the axillary vein has corresponds to low wall sheer stress within the cephalic arch, promoting intimal hyperplasia and hypertrophic remodeling^13,14^. Since CAS lesions are generally short and focal in nature, they are associated with increased morbidity and may lead to high venous pressures, prolonged bleeding after dialysis, dysfunctional dialysis, increased rate of fistula thrombosis, and fistula failure.

Despite being the most common stenosis in dysfunctional AVAs, accounting for 30% to 55% of all upper arm access stenosis^3^, CAS is still known to be notoriously difficult to treat and responds poorly treatment by venoplasty alone, only providing primary patency rate of 42% at 6 months^15^. Treatment of CAS is further complicated by higher rupture rate due to increased pressure required for anatomical success. As venoplasty alone has not demonstrated significant lasting benefits for CAS, recent comparison has demonstrated superiority of SG over other modalities of endovascular treatment, specifically venoplasty, BMS and drug-eluting stent in the treatment of CAS, at maintaining CAS patency^7^.

Although SGs has demonstrated significant benefits over other modalities of treatment of CAS, common occurrence of edge stenosis has prevented SG from becoming the standard treatment for CAS. One previous study has reported the primary stenosis site was observed mostly at the lateral edge of the SG, accounting for 52% of stenosis episodes. Other stenosis site included bilateral edge stenosis, medial edge stenosis and finally in-stent stenosis only occurred in 29%, 17%, and 2% respectively of all SG related stenosis^10^.

We also documented rapid onset of lateral edge stenosis in our patients after SG placement, especially in patients with SG diameters similar or greater than the adjacent cephalic vein. We noticed among patients with SG smaller than the adjacent cephalic vein, reintervention for stent failure and edge stenosis was less frequent. As a result, our study retrospectively recruited all patients with SG placement at the cephalic arch to compare the patency rate of SG of the patients whose SG are apposed to the cephalic vein with those with SG undersized relative to the cephalic vein.

The two groups were comparable in clinical characteristics (Table 1). Furthermore, the prevalence of cephalic arch stenosis among those with brachiocephalic AVF and radiocephliac AVF was similar to a previously reported study^5^. Among the patients in the undersized SG group, smaller stents were used resulting in significantly smaller S/V ratios. Outcomes showed a significant advantage for using undersized SG over apposed SG: patients with undersized SG required a decreased amount of post-SG interventions needed per access-year for AVA dysfunction, decreased percentage of lateral edge stenosis, and a higher primary stent patency rate and primary access patency rate.

While SGs has been used to great effect in the treatment of arterial related diseases such as aortic aneurysms, peripheral arterial diseases, and pseudoaneurysms, research trials and guidelines suggest oversized SG to reduce SG migration. However, the nature and structure of veins are known to be vastly different from arteries, and SGs specifically designed for use in arteries may not be ideal for treatment of venous lesions such as refractory stenosis or venous vessel rupture^16^. To the best of our knowledge, there have been no previous studies discussing proper SG sizing for use in AVAs, whereas undersized SG has been previously shown in Gore REVISE Clinical Study to improve primary stent patency rate^17^. Without adequate research, the guidelines of oversizing SG may be suboptimal when treating cephalic arch stenosis or other venous stenosis^16^.

While the majority of SG failure in the cephalic arch is currently attributed to neointimal growth causing edge stenosis at the junction of SG and the apposed vessel wall^8,18,19^, the exact cause of neointimal growth has not been adequately investigated. Previous fluid dynamic studies have shown that apposition of SG to adjacent vessel wall may lead to higher shear stresses at the outflow vein, and can lead to development of neointimal hyperplasia, resulting in edge stenosis^20,21^. Undersized SGs minimize the contact between the medial and lateral edges of the SG to adjacent venous wall and may help reduce neointimal hyperplasia at the edge of the SG and possibly prevent future edge stenosis.

Stent migration is a common complication associated with undersized stent placement, and is typically remedied by selection of oversized stents. Despite placing smaller SG in our undersized patient group, there was no evidence of stent migration at the last angiographic follow up for each of the 22 patients, nor were there any symptoms or treatments related to stent migration. We believe that due to the tortuous anatomy of the cephalic arch, SG are able to maintain their position despite being relatively undersized compared to both the cephalic and subclavian vein. Furthermore, venous stenosis at the cephalic arch allows the SG to be fixed in place via apposition of the central portion of the stent to the stricture location. Among the 12 patients with ruptured vessels due to balloon dilation, adequate extravasation control was noted in all patients, despite undersizing SG in 7 of the 12 patients. Control of extravasation may be maintained with undersized SG due to two reasons. Firstly, SG were chosen such that the diameter is equal or greater than diameter of the balloon that caused vessel rupture during prior balloon assisted angioplasty. Secondly, although the SG is undersized relative to the medial and distal vessel, it maintains apposition to the stenosis site, and allows for adequate extravasation control.

Another concern of undersized stent placement is the increased flow resistance and wall stress at the inflow area caused by tiny gaps between the stent and the wall in arteries^22^. However, we detected no evidence that under sizing SG caused significant flow disturbances in our cohort resulting in venous hypertension, poor hemodialysis function, or other clinical symptoms. It is possible that the relatively slow flow rate in the vein decreases the impact of the flow turbulence on stent stenosis. Among the 22 patients, no events of elevated venous pressure were directly caused by the undersized stent. Rather, edge or in-stent stenosis were the main culprit in patients presenting with high venous pressure after SG placement.

Viabahn Endoprosthesis was used in all cases in this study and was selected for its flexibility and ability to conform to the arch of the cephalic vein. When placed in the tortuous vessels such as the cephalic arch, the natural anatomy is maintained and avoids “tenting” of the cephalic arch^23^. In addition, full covering of the expanded polytetrafluoroethylene liner over the external metal nitinol structure reduces rate of in-stent and edge stenosis^24^. The unique mechanical properties of the Viabahn Endoprosthesis appear to prolong the patency time in the treatment of CAS^23^.

Optimal deployment of SG is another important factor in maximizing the patency rate after treatment of CAS. At our hospital, SGs are deployed across the entire length of any diseased cephalic vein and the cephalic arch with the medial end of the SG protruding into the subclavian vein. Covering this length reduces the likelihood of stenosis in segments of the cephalic arch known to have increased stenosis risk^25^. However, accurate SG placement at the cephalic arch is especially difficult due to the angle of entry and the anteroposterior orientation of the confluence into the axillary vein. By protruding the SG into the subclavian, we not only ensure that the SG covers the entire cephalic arch, but also lowers the difficulty of positioning of the SG when using angiography alone. Another possible advantage of extending the stent into the subclavian vein is the reduction of turbulent flow at the junction of the cephalic arch and the subclavian vein through alignment of the returning flow vectors^23^. Operators should remain aware that SG protrusion into the subclavian vein may increase risk of central vein occlusion^10^, even though, we observed no symptoms of central vein occlusion, including high venous pressure or arm swelling. A secondary benefit of selecting relatively smaller SGs may be reduced obstruction of the returning axillary venous flow, thus mitigating risk of stenosis and occlusion.

To the best of our knowledge, there has been no previous comparative study on the optimal SG diameter for treatment of CAS. Although oversized SG is recommended for treatment of arterial disease to prevent SG migration, oversizing SG may be suboptimal for treatment of CAS or other venous diseases. There should be more considerations when using arterial devices for treatment of venous pathologies.

A number of limitations should be noted in this study, including the reliance of previously collected data for this retrospective study. Second, there is still a relatively low number of patients and may reduce the power and increases the margin of error of this study. Further research with increased patient size and randomized control groups may be necessary to strengthen our study findings. Lastly, symptomatic follow-up through venoplasty and venography potentially underestimates patency rates in subclinical stenosis.

Undersized SG in patients with CAS showed significant higher primary stent and access patency rates, lower number of post-SG interventions per access-year, and lower number of lateral edge SG stenosis compared to patients with apposed SG. The present study emphasizes the need for more deliberations when sizing SG for treatment within AVAs, and future prospective trials comparing the placement of undersized SG verses apposed SG should be done to determine which improves patency.

## Data Availability

The datasets generated during and/or analyzed during the current study are available from the corresponding author on reasonable request.
